# Broadband Spectra and Efficient Laser Performance of a Disordered Nd:Sr_0.7_La_0.3_Mg_0.3_Al_11.7_O_19_ Crystal

**DOI:** 10.3390/ma11091575

**Published:** 2018-09-01

**Authors:** Nana Zhang, Xiaodong Xu, Yuxin Pan, Jian Liu

**Affiliations:** 1 College of Chemistry & Chemical and Environmental Engineering, Weifang University, Weifang 261061, China; 2 Jiangsu Key Laboratory of Advanced Laser Materials and Devices, School of Physics and Electronic Engineering, Jiangsu Normal University, Xuzhou 221116, China; yuxinpan132@163.com (Y.P.); jianliu132@163.com (J.L.)

**Keywords:** Nd:ASL, Czochralski method, Judd–Ofelt theory, CW, passively Q-switched

## Abstract

Here, we demonstrate the broadband spectra and efficient laser performance of a disordered Nd:Sr_0.7_La_0.3_Mg_0.3_Al_11.7_O_19_ (Nd:ASL) crystal. The polarized absorption and emission spectra were characterized and calculated with the Judd–Ofelt (J–O) theory, and the results show that this crystal has relatively large σ polarized absorption and emission cross sections, with peak emission cross sections of 4.5 × 10^−20^ cm^2^ at 1051 nm and an emission width of 30 nm at 1074 nm, which are favorable for pulsed lasers. The laser-diode-pumped continuous-wave (CW) and passively Q-switched lasers at 1051 nm were realized for the first time to our best knowledge. For CW laser, the maximum output power was 1.95 W with the slope efficiency of 42.6%, and for the passive Q-switching, the maximum output power was 0.73 W with the pulse width of 7.96 ns and peak power of 5.64 W. The results demonstrate that Nd:ASL crystal is a potential candidate for lasers, especially high-peak pulsed lasers such as Q-switching and mode-locking.

## 1. Introduction

Diode-pumped pulsed lasers have been investigated and used widely in many applications of biotechnology, medical surgery, telemetry, etc. [1,2,3,4,5]. Types of pulsed lasers include mode-locking and Q-switching, which favor broad emission spectra for ultrashort pulses and small emission cross sections for large energy-storage properties. Based on the relationship between the gain and width of emission spectra [6], we can find that the laser crystals with broad spectra should have small emission cross sections and large energy-storage properties. Therefore, the investigation of laser crystals with broad spectra is critical for pulsed lasers. Nd^3+^-doped materials normally have narrow spectra, e.g., the full width at half maximum (FWHM) of Nd:YAG is 0.5 nm, and the FWHM of Nd:YVO_4_ is about 1.5 nm. Therefore, previously, favorable pulsed laser gains were focused on Yb^3+^-doped crystalline materials, including crystals and ceramics [7]. However, the Nd^3+^-doped materials have advantages in peculiar four-level systems, which means that these lasers have a much lower laser threshold compared with those of Yb^3+^-doped materials [8,9,10]. Thus, it is interesting to investigate the host materials which can broaden the spectra of Nd^3+^ ions. In theory, the spectra of active ions can be homogeneously and inhomogeneously broadened by introducing a disordered structure, and inhomogeneous broadening can play important roles. A successful material with inhomogeneously broadened spectra is Nd:glass, the mode-locked laser of which reaches 38 fs, but it is limited by its small thermal conductivity as a result of the disordered structure, and the output power is only 40 mW [11]. Due to the crystalline structures, crystals usually have advantages in thermal conductivity over glass. In recent years, disordered crystals have emerged and attracted more and more attention because of their disordered structure in coordinating polyhedrons. Plenty of disordered crystals, including Nd:CNGG, Nd:CALGO, Nd:CLB, etc., have been reported [12,13,14], and in 2016, Ma et al. achieved stable continuous-wave (CW) mode-locked pulses as short as 79 fs in a diode-pumped Nd:CLB disordered crystal laser [14]. 

Sr_1−x_Nd_y_La_x−y_Mg_x_Al_12−x_O_19_ (Nd:ASL) is a disordered laser crystal for which Sr^2+^ ions are partially replaced simultaneously by La^3+^ and Nd^3+^ and Al^3+^ ions by Mg^2+^ in SrAl_12_O_19_. Both Mg^2+^ and Al^3+^ exhibit similar ionic radii (r(Mg^2+^) = 66 pm, r(Al^3+^) = 54 pm), and when Mg^2+^ ions get into the lattice of the Al^3+^ ion, the disordered microstructure resulting from the charge imbalance between Mg^2+^ and Al^3+^ leads to an inhomogeneous broadening of the absorption and emission spectra of a Nd-doped strontium and lanthanum alumninate crystal. Further, the ASL crystal possesses a magnetoplumbite structure with a P6_3_/mmc space group [15,16] and has the advantage of good corrosion resistance. In previous works, the study of this crystal focused on flash-pumped lasers at about 900 nm, while laser-diode-pumped solid-state lasers over 1 μm were little reported [16,17]. In 2018, S. Sattayaporn et al. have also reported 267, 52, and 318 mW continuous laser output in Pr^3+^-doped ASL crystals [18]. 

In the present work, we mainly report on the promising spectral and laser properties of the Nd:ASL (Sr_0.7_Nd_0.05_La_0.25_Mg_0.3_Al_11.7_O_19_) crystal, including the absorption and emission spectra and continuous-wave and passively Q-switched lasers. All the results indicate that the Nd:ASL crystal should be a potential candidate for the generation of pulsed lasers. 

## 2. Results and Discussion

### 2.1. Spectral Characterization of the Nd:ASL Crystal

The Nd:ASL crystal with 5 at.% Nd^3+^ ion doping was grown using the Czochralski method. The raw materials for growing the crystal were Nd_2_O_3_, SrCO_3_, La_2_O_3_, Al_2_O_3_, and MgO with 99.999% purity, respectively, which were firstly dried, weighed, and mixed according to the formula Sr_0.7_Nd_0.05_La_0.25_Mg_0.3_Al_11.7_O_19_. After mixture, they were pressed into pellets and then sintered at 1300 °C for 20 h in the air to form the Nd:ASL polycrystalline material. Crystal growth was carried out in an iridium crucible with an intermediate frequency furnace. An a-axis LaMgAl_11_O_19_ rod (Φ 6 mm × 45 mm) was used as the seed. The pulling rate was 1 mm/h after the crystal diameter reached a certain value, and the rotation rate was 15 rpm. High-purity nitrogen gas was introduced as a protective atmosphere. After growth, the crystal was cooled to room temperature slowly at a speed of 30–45 °C/h. The as-grown Nd:ASL crystal boule was clear with blue color and free from cracks, inclusions, and scattering centers. 

**Figure 1:**
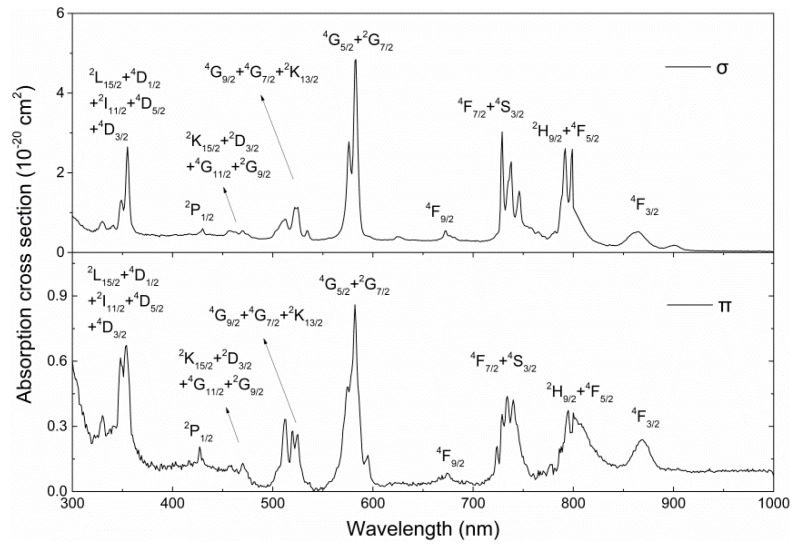
Polarized absorption cross-section spectra of a Nd:Sr_0.7_La_0.3_Mg_0.3_Al_11.7_O_19_ (Nd:ASL) crystal at room temperature.

The polarized absorption spectra were measured by a JASCO (Kyoto, Japan) model V-570 UV/VIS/NIR Spectrophotometer V-570 at room temperature. The polarized absorption cross section of the Nd:ASL crystal is shown in Figure 1 in the region of 300–1000 nm at room temperature. Due to the anisotropy of this crystal, the absorption spectra had strong polarization dependence. We can see that the three strong bands around 583, 729, and 793–798 nm correspond to the transitions ^4^I_9/2_→^4^G_5/2_ + ^2^G_7/2_, ^4^I_9/2_→^4^F_7/2_ + ^4^S_3/2_, and ^4^I_9/2_→^4^F_5/2_ + ^2^H_9/2_, respectively, and the σ-polarized absorption is stronger than that along π polarization. The absorption cross sections for σ and π polarizations are 2.6 × 10^−20^ cm^2^ at 792 nm and 0.37 × 10^−20^ cm^2^ at 795 nm, with the FWHM of 12 and 37 nm, respectively. Broadband absorption is well-adapted for efficient diode-pumping with commercially available high-power AlGaAs laser diodes.

**Figure 2:**
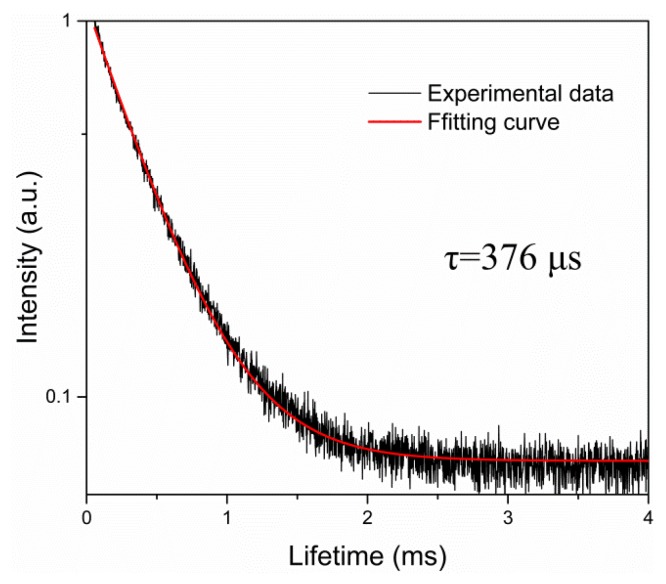
Room temperature fluorescence decay curve of the 4F_3/2_ manifold of the Nd:ASL crystal.

Fluorescence spectra and fluorescence decay curve at 1051 nm were recorded by a FSP980 spectrometer (Optosci, Edingurgh, UK) under 796 nm excitation. The polarized fluorescence spectra of the Nd:ASL crystal was obtained under irradiation of a laser diode with the wavelength of 800 nm. The fluorescence decay curve (as shown in Figure 2) of the ^4^F_3/2_→^4^I_11/2_ energy transition was measured with the lifetime of 376 µs, which is much longer than that of the Nd:SrLaGa_3_O_7_ crystal, which is a disordered crystal-laser crystal for femtosecond pulsed lasers [19]. The results show that the Nd:ASL crystal is a promising medium for solid-state lasers. 

The Judd–Ofelt (J–O) theory was applied in our work, which is the most popular and useful method to assess spectra parameters of rare earth ions. We followed similar calculation procedures as those reported in [20], and the average wavelength of the different transitions, the oscillator strength of the experiment and calculations, and the RMS (root mean square error caused by spectral parameters through J–O theory) deviation of the Nd:ASL crystal are given in Table 1. The value of RMS is 0.16 × 10^−20^ cm^2^ for π polarization and σ polarization, which indicates good agreement between the experimental spectral intensities and the calculated intensities.

J–O intensity parameters Ω_2_, Ω_4_, and Ω_6_ are given in Table 2. The effective intensity parameters were calculated by Ω = (2Ω_σ_ + Ω_π_)/3, and the Ω_2,4,6_ were found to be 1.10, 3.48, and 2.96 × 10^−20^ cm^2^, respectively. The spectroscopic quality Ω_4_/Ω_6_ of the Nd:ASL crystal for σ polarization was 1.18 (more than 1), which indicates that the emission to the ^4^I_9/2_ manifold is more feasible than that to ^4^I_11/2_ manifold. All of the results fit well with the emission spectra. The spontaneous transition rate, branching ratios, and the radiative lifetime of ^4^F_3/2_→^4^I_9/2_, ^4^F_3/2_→^4^I_11/2_, ^4^F_3/2_→^4^I_13/2_, and ^4^F_3/2_→^4^I_15/2_ transitions of the Nd:ASL crystal are given in Table 3. The branching ratio of the ^4^F_3/2_→^4^I_9/2_ transition is smaller than that of the ^4^F_3/2_→^4^I_11/2_ transition for σ polarization, and the radiative lifetime of the ^4^F_3/2_ energy level was calculated to be 501 µs.

**Figure 3:**
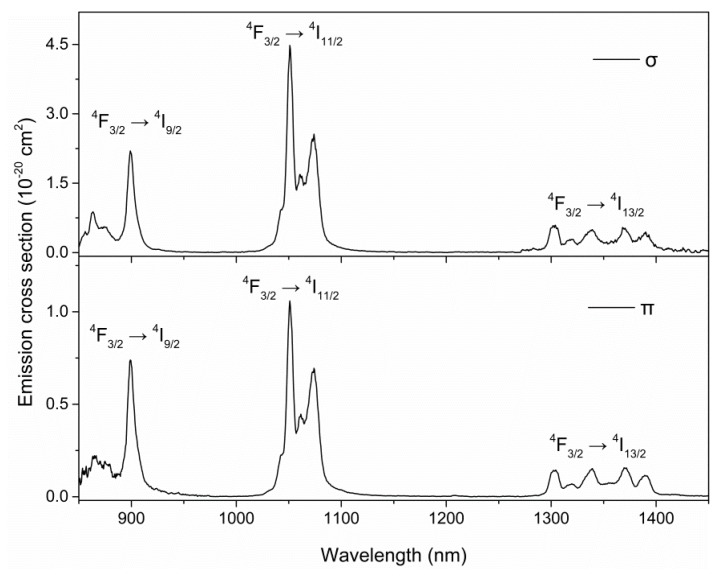
Stimulated emission cross section of Nd:ASL crystal excited by 800 nm at room temperature.

The stimulated emission cross sections calculated from the fluorescence spectra using the J–O theory [21,22] are shown in Figure 3. Three emission bands, ^4^F_3/2_→^4^I_9/2_, ^4^F_3/2_→^4^I_11/2_, and ^4^F_3/2_→^4^I_13/2_, which were centered at 850–940, 1040–1160, and 1320–1440 nm, were assigned, respectively. The peak emission cross section is 4.5 × 10^−20^ cm^2^ for σ polarization and 1.1 × 10^−20^ cm^2^ for π polarization, both at 1066 nm and with FWHM of 30 and 32 nm, respectively. The results show that this crystal should have promising applications in pulsed lasers, including Q-switching and mode-locking.

### 2.2. Continuous-Wave and Passive Q-Switched Lasers of Nd:ASL Crystal

**Figure 4:**
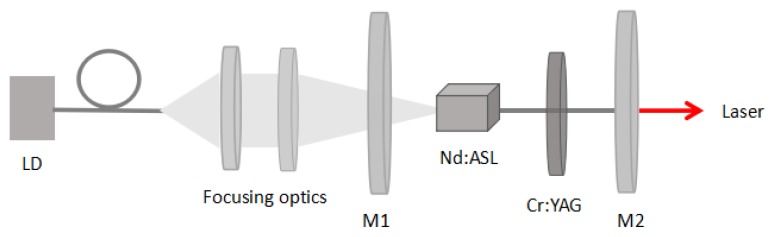
Continuous-wave (CW) and Q-switched laser setup of the Nd:ASL crystal.

As shown in Figure 4, the CW and passive Q-switched Nd:ASL lasers were realized in stable plano-concave and planoplano cavities, respectively. The pump source was a fiber-coupled laser diode (LD) at the emitting central wavelength of 795 nm. The sample was cut with the dimensions of 3 × 3 × 5 mm^3^ (a × a × c) to obtain the σ-polarization emission. Both end faces were polished and antireflection (AR) coated in the condition of the lasing wavelength at 1064 nm and the pumping wavelength at 795 nm. The output beam of the source was focused onto the Nd:ASL crystal sample by the focusing optics. In a plano-concave cavity, M1 was a flat mirror, AR was at 795 nm, and high-reflective (HR) was at 1060 nm. M2 was a concave output coupler with a radius of curvature of 50 mm with different output transmissions of 1%, 3%, and 5% at 1060 nm, respectively. The lengths between M1 and M2 were optimized during the experiments. To eliminate the heat produced from the laser crystal in the experiments, the sample was bound with indium foil and cooling water was maintained at a temperature of 13 °C. For the Q-switched laser experiment, a Cr^4+^:YAG crystal (thickness of 0.5 mm) was inserted between M2 and the Nd:ASL crystal. The initial transmission of the Cr^4+^:YAG crystal was 80%. 

**Figure 5:**
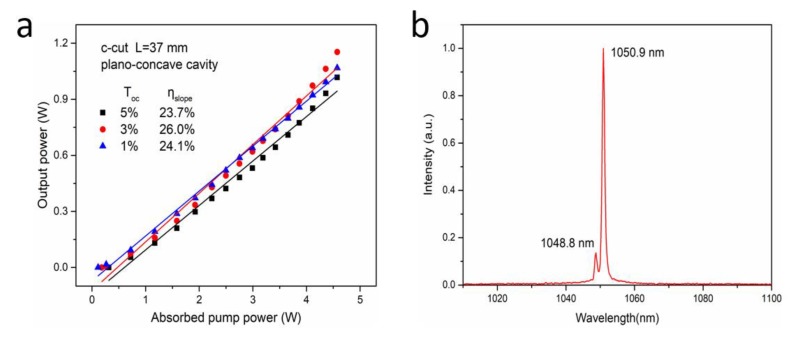
The output performance of Nd:ASL lasers with a laser cavity of 37 mm (**a**) and the laser spectra (**b**).

The CW laser operation of the Nd:ASL crystal was investigated in a plano-concave cavity. The output of the laser was characterized to be σ polarized. Figure 5a shows the CW laser characteristics of the Nd:ASL laser crystal, which were obtained under different output couplers. By optimizing the cavity length (L) to be 37 mm, the thresholds of the CW lasers were optimized to be 0.11, 0.18, and 0.31 W when the transmissions of the output couplers were 1%, 3%, and 5%, respectively. Under this cavity length, when the output coupler was 3% and the absorbed pump power was 4.57 W, we obtained the highest output power of 1.15 W, which corresponded to that of an optical conversion efficiency of 25.2% and a slope efficiency of 26.0%, respectively. The output laser spectrum is shown in Figure 5b. From this figure, we can see that the output wavelength is a dual-wavelength at 1048.8 and 1050.9 nm, which correspond to the spontaneous peak emission cross sections. With the knife-edge method, the continuous-wave laser beam quality was measured and produced a result of 1.3.

**Figure 6:**
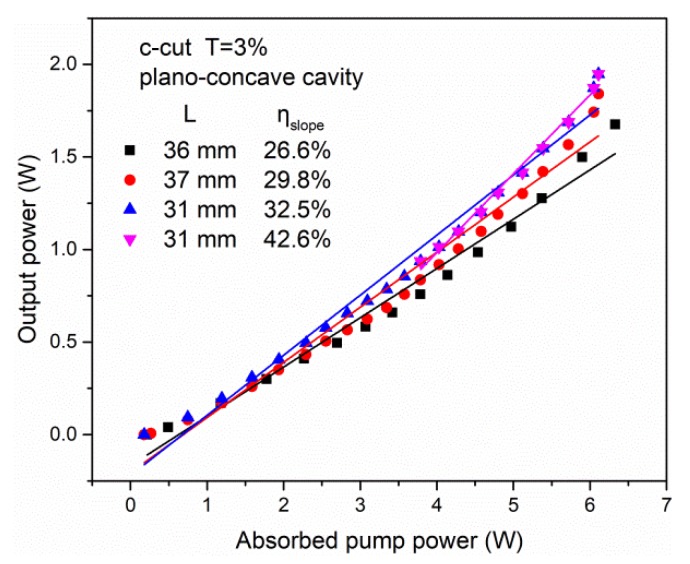
The CW laser characteristics of the Nd:ASL crystal with different cavity lengths along the c-axis.

With the optimized output coupler of T_oc_ = 3%, the cavity length was optimized to achieve efficient laser output because the thermal effects, especially the thermal lens, would influence the stability of the cavity. The output performance is shown in Figure 6. With the ABCD matrix, we can see that the optimized cavity length should be reduced with the increase of the incident pump power. Considering the threshold, we optimized the cavity length to 31 mm, and the slope efficiency was 32.5%. For this condition, we also optimized the focusing position in the crystal associated with the efficiency and obtained the highest slope efficiency of 42.6%. We also found that the Nd:ASL laser was σ polarized.

Some optical parameter values of the Nd:ASL crystal are listed in Table 4, in which we also listed data from other laser materials for comparison. Table 4 shows that the Nd:ASL crystal possesses small emission cross sections, which indicate that it should have a higher energy-storage capacity and would require a well-designed cavity for mode-locking.

**Figure 7:**
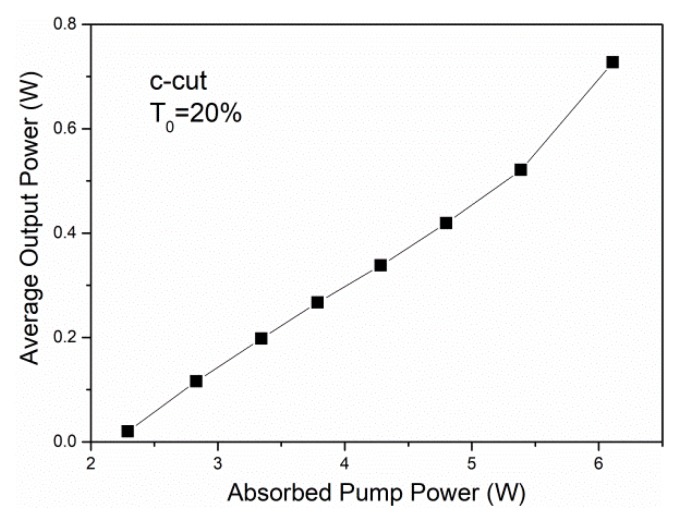
Passively Q-switched laser output of the Nd:ASL crystal.

Inserting the Cr^4+^:YAG crystal with a transmission of 80% into the cavity, we can achieve passive Q-switching. In order to avoid damage in the cavity, the output coupler with a transmission of 20% was employed. In order to reduce the beam radius in the Cr^4+^:YAG crystal, the saturable absorber was located close to the output coupler. The average output power performance is plotted in Figure 7. With an optical conversion efficiency and a slope efficiency of 11.9% and 17.5%, respectively, the threshold pump power was measured to be 2.29 W and the maximum average output power was 0.73 W, respectively. 

**Figure 8:**
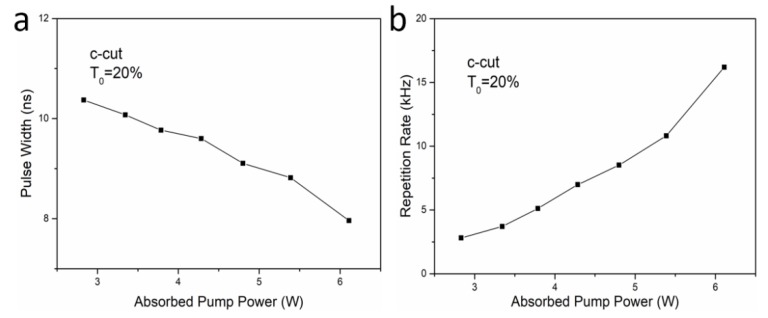
Pulse width (**a**) and repetition rate (**b**) versus the absorbed pump power for the passively Q-switched Nd:ASL laser.

The pulse width and the repetition rate were recorded by an oscilloscope and a photodetector, and the results obtained are illustrated in Figure 8a,b, respectively. From this figure, we can find that the pulse width decreased obviously at the augmentation of the absorbed pump power, while the repetition rate increased. When the absorbed pump power was 6.11 W, we found that the minimum pulse width was 7.96 ns. From 2.81–16.19 kHz, the repetition rate increased almost linearly with the pump power. With the repetition rate and average output power, the maximum pulse energy calculated was 44.9 μJ. With the pulse width, the maximum pulse peak power calculated was 5.64 kW. The output laser was also found to keep σ polarization. We believe that the passive Q-switching performance can be highly improved by optimizing the cavity, including the transmission of the output coupler and initial transmission of Cr:YAG saturable absorbers. 

## 3. Conclusions

In conclusion, the spectra and laser performance of a disordered Nd:ASL crystal were investigated. Polarized absorption and fluorescence spectra were measured at room temperature. By the J–O theory, the absorption and emission cross sections for σ polarization were calculated with the peak absorption cross section of 2.6 × 10^−20^ cm^2^ at 792 nm and FWHM of 12 nm and the emission cross section of 4.5 × 10^−20^ cm^2^ and FWHM of 30 nm, respectively. We demonstrated the CW and passively Q-switched laser operation of the Nd:ASL crystal. The highest CW output power of 1.95 W was obtained in a plano-concave cavity, corresponding to a slope efficiency of 42.6%. For passively Q-switched operation, the shortest pulse, maximum pulse energy, and the highest peak power were 7.96 ns, 44.9 μJ, and 5.64 kW, respectively. The results show that Nd:ASL should be an excellent disordered crystal with promising applications in pulsed lasers, especially in Q-switching and mode-locking lasers, which will be further studied.

## Figures and Tables

**Table 1:** The average wavelength of different transitions, the line strength of the experiment and calculations, and RMS (root mean square error caused by spectral parameters through J–O theory) deviation of Nd:ASL crystal.

Excited State	σ Polarization			π Polarization		
^4^I_9/2_	(nm)	(10^−6^)		(nm)	(10^−6^)	
	λ	S_exp_	S_cal_	λ	S_exp_	S_cal_
^2^L_15/2_ + ^4^D_1/2_ + ^2^I_11/2_ + ^4^D_5/2_ + ^4^D_3/2_	349	2.834	2.661	347	1.025	0.954
^2^P_1/2_	433	0.088	0.162	437	0.059	0.060
^2^K_15/2_ + ^2^D_3/2_ + ^4^G_11/2_ + ^2^G_9/2_	465	0.576	0.340	465	0.162	0.106
^4^G_9/2_ + ^4^G_7/2_ + ^2^K_13/2_	518	1.693	1.565	517	0.662	0.492
^4^G_5/2_ + ^2^G_7/2_	580	4.389	4.350	580	1.311	1.305
^4^F_9/2_	676	0.211	0.210	676	0.066	0.054
^4^F_7/2_ + ^4^S_3/2_	740	3.036	2.823	738	0.739	0.682
^2^H_9/2_ + ^4^F_5/2_	797	2.931	3.133	806	0.812	0.876
^4^F_3/2_	869	1.103	1.228	873	0.260	0.425
RMS (10^−20^ cm^2^)	0.182			0.110		

**Table 2:** The J–O intensity parameters of the Nd:ASL crystal.

Intensity Parameter	Ω (10^−20^ cm^2^)		
	σ-Polarization	π-Polarization	Ω = (2Ω_σ_ + Ω_π_)/3
Ω_2_	1.51	0.28	1.10
Ω_4_	4.40	1.63	3.48
Ω_6_	3.98	0.92	2.96

**Table 3:** The spontaneous transition rate, branching ratios and radiative lifetime for different transition levels of Nd:ASL crystal.

Transitions	σ Polarization		π Polarization	
	A_σ_ (S^−1^)	β_σ_ (%)	A_π_ (S^−1^)	β_π_ (%)
^4^F_3/2_→^4^I_9/2_	1623.75	45.43	557.48	52.57
^4^F_3/2_→^4^I_11/2_	1636.06	45.77	430.21	40.57
^4^F_3/2_→^4^I_13/2_	308.14	8.62	71.20	6.71
^4^F_3/2_→^4^I_15/2_	6.61	0.18	1.53	0.14
Radiative lifetime (µs)	τ_rad_ = 501			

**Table 4:** Optical parameter values of some laser materials.

Crystals	Properties			
	Emission Cross Section (10^−20^ cm^2^)	Fluorescence Bandwidth (nm)	Lifetime (μs)	Continuous-Wave Output Power (W)
Nd:CNGG [12]	5 (1059)	15	210	3.9
Nd:CaYAlO_4_ [20,23]	7.53 (π), 10.44 (σ)	15 (π), 12 (σ)	129	5.16
Nd:SrLaGa_3_O_7_ [19,24]	10	14	310	1.16
Nd:CaGdAlO_4_ [25]	0.75 (σ)	80 (σ)	420	0.36
Nd:ASL (present work)	4.5 (σ), 1.1 (π)	30 (σ), 32 (π)	376	1.95
